# Promoting a Safe Environment in Our Cities: Towards a Theoretical Model of “Moral Deficit” for Appropriate Psychopathic Therapy

**DOI:** 10.3390/ijerph17144968

**Published:** 2020-07-10

**Authors:** David Coldwell, Sarah Coldwell

**Affiliations:** 1 School of Business Sciences, Faculty of Commerce, Law and Management University of the Witwatersrand, Johannesburg 2050, South Africa; 2 Welfare Services, Electrical Industries Charity in London, London SW19 8SE, UK; scoldwell90@gmail.com

**Keywords:** psychopaths, moral deficit, therapy, terrorism, safe environment

## Abstract

The increasing reported incidents of knife crime in cities and the release on parole of “rehabilitated” violent criminals are creating an unsafe urban environment. Such occurrences suggest that measures taken to address psychopathic-oriented behaviour may have been ineffective because the individual’s degree of “moral deficit” is not fully accounted for in the application of specific therapies. This study developed a theoretical model of “moral deficit” that is aligned with the appropriateness of therapy, ranging from the extreme “classical approach” of total confinement justified by a belief in the incurability of psychopaths to the modern therapy that aims to reintegrate the psychopath with society using “moralizing therapy”. Analysis of secondary data from extant literature was used to develop the theoretical model of “moral deficit”. Secondary data analysis suggests that the extent of psychopathic “moral deficit” may be an important factor in the selection of appropriate therapeutic measures for psychopathy treatment and the rehabilitation of psychopaths as law-abiding members of society. We conclude that a specific type of psychopathic moral deficit may have an important bearing on the appropriateness of treatment. It is recommended that the treatment of psychopathy makes greater provision for the extent and type of psychopathic “moral deficit” in assessing the most appropriate applications for the treatment of psychopathy and promoting the safety of urban environments.

## 1. Introduction

The increasing reported incidents of knife crime in cities and the release on parole of “rehabilitated” violent criminals are creating an unsafe urban environment. Such occurrences suggest that measures taken to address psychopathic-oriented behaviour may have been ineffective because the individual’s degree of “moral deficit” is not fully accounted for in the application of specific therapies. This study developed a theoretical model of “moral deficit” that is aligned with the appropriateness of therapy, ranging from the extreme “classical approach” of total confinement to the modern therapy that aims to reintegrate the psychopath with society using “moralizing therapy”. Analysis of secondary data from extant literature was used to develop the theoretical model of “moral deficit”. Secondary data analysis suggests that the extent of psychopathic “moral deficit” that specifically combines high moral development with an unbalanced integration of idealism and relativism in an individual’s moral outlook may be an important factor in the selection of appropriate therapeutic measures for psychopathy treatment and rehabilitation, as well as a timely release back into society. The paper takes the following form. Section 2 discusses the background to the study, which is divided into two subsections, where Section 2.1 and Section 2.2 discuss the theoretical aspects and Section 2.3 discusses the practical aspects of the proposed exploratory model. Section 2 is followed by a discussion of the methodology of the secondary data analysis, which incorporates directed content analysis of eclectic historical narrative, used in the study. In Section 3, the results of the eclectic secondary data material selection are described. This is done in two subsections. The first subsection describes the eclectic secondary data drawn from a potentially broad base of diverse sources of specific psychiatric and psychological reports, and media historical narratives of violent psychopathic behaviour are then described and discussed relative to the theoretical aspects of postulated moral deficit exploratory model. The second subsection focuses on the practical problem of psychopathy relative to the effectiveness of therapy and the prospects of rehabilitation among violent recidivistic psychopaths. Section 4 describes and explains the exploratory model in terms of the secondary case study data and extant findings on psychopathic violence, recidivism and prospects of rehabilitation discussed earlier in the paper. Section 5 presents the discussion section of the paper, which is followed by the conclusion in Section 6, which presents an overview of the exploratory model, its limitations and recommendations for further research.

## 2. Background of the Study 

The background of the study is divided into subsections, with Section 2.1 and Section 2.2 discussing the theoretical aspects and Section 2.3 discussing the practical aspects of the proposed model.

### 2.1. Theoretical Aspects: The Concept of Moral Deficit

Forsyth [1,2,3,4] created an interactional model to address differences in the way individuals make moral judgments. The way an individual assesses an ethical dilemma expresses their integrated system of ethics and is evidence of that person’s “individual moral philosophy” (IMP). An individual’s moral beliefs, values and attitudes comprise the IMP, which “provides guidelines for moral judgments, solutions to ethical dilemmas and prescriptions for actions in morally toned situations.” [1]. Individual variations in IMP are based on two basic factors: relativism and idealism [5]. Forsyth [1] defined idealism as the extent to which individuals “assume that desirable consequences can, with the right action, always be obtained”. Relativism is defined by Forsyth [1] as, “the extent to which an individual rejects universal moral rules” when making moral judgments. Forsyth [1] maintains that differences in relativism and idealism influence individual moral judgment; idealistic persons shun beliefs and activities that harm or impact negatively on others, while relativistic persons feel that moral actions are dependent on specific moral situational dilemmas that confront an individual, and that appropriate moral judgments consider the situation to be more than any ethical principles that may be violated. For Forsyth [1], either idealism or relativism dominate moral judgement and characterises the individual as being either one or the other. However, the possibility of idealism and relativism occurring together and emerging in a singular moral edifice such that high idealism and relativism occur in the same individual or individuals with the same beliefs is not specifically considered by Forsyth [1]. A very clear example of this phenomenon of idealism and relativism occurring interactively is found in Nazi ideology and ethics evidenced in the notoriously evil SS (Hitler’s “Shutz Staffel”, who were part of the Waffen SS militia responsible for running the death camps and the elimination of the Jews). In this regard, Bialas and Fritze [6] indicate that when Nazis considered the Jews:

*SS ethics combined deontological, consequentialist and perfectionist approaches organized around moral concepts such as duty, the good and virtuousness while at the same time bereaving these concepts of their universal nature. … [T]his way of restricting the common good to one people legitimated every kind of violence, after all. By attributing value only to part of humanity, SS ethics pursued excessive egotism, thus at the same time showing a strong nihilistic component.*

The mix of idealism and relativism in Nazi ideology is most horrifically portrayed by Himmler, (Reichfuhrer-SS) in his speech to SS personnel at Posen in 1943 [7]:

*We had the moral right, we had the duty towards our people to kill this people which wanted to kill us. But we do not have the right to enrich ourselves with so much as a fur, with a watch, with a Mark or with a cigarette or anything else.*

Wachsmann [7] goes on to point out that this was no idle threat on the part of Himmler, who showed no mercy to SS personnel who had transgressed the universal moral ideal of honesty (thou shalt not steal) with a relativistic conception of duty, which went only so far as preserving Aryan lives while advocating the extermination of Jews.

Mineau [8] makes a similar point about Henrich Himmler:

*Heinrich Himmler is mostly seen as the all-powerful organizer who coordinated the police apparatus that reigned over occupied Europe and who personally supervised the concentration camp system. But Himmler was also a thinker or, at least, he perceived himself as such, and he was especially concerned with moral issues. … From a normative viewpoint any claim to philosophical validity for this type of approach may be called into question, for the nihilism and denial of otherness were paramount in the instrumentalization of humans that led to genocide.*

This thinker element to Himmler’s moral framework suggests that despite his aberrant idealism–relativism mix, he had a high level of moral development. We are not suggesting that Himmler was a psychopath (although it is not inconceivable that he was) applying the multifaceted definitions and measures used today for that dubious distinction, but only that he displayed a specific form of “moral deficit” that we suggest in the paper may be a factor in violent criminal psychopathic-type behaviour. Perhaps the most celebrated theory of moral development that has been subjected to extensive empirical testing is that devised by Kohlberg [9], which for the current paper, requires an outline description.

Kohlberg’s [9] theory of moral development indicates that there are three major stages in moral development. The “pre-conventional stage” describes the earliest stage of moral development; it describes the most basic level of moral development found in very young primary school children. At this basic stage of moral development, moral behaviour emphasises obedience with lapses enforced by punishment. Later in the pre-conventional stage of moral development, young children develop an individualist and instrumentalist attitude towards interpersonal exchange and their interaction with the world. This further development at this stage of moral development is sometimes referred to as the ‘‘seeking-of-rewards stage’’ [10], which is further developed in the next major Kohlbergian stage he describes as the “conventional stage”. Kohlberg [9] suggests that the conventional stage of moral development is, normatively speaking, the “final” stage for most adults. The conventional level is divided into two separate substages. The first substage is characterised by ‘‘good boy/nice girl’’ approval-seeking behaviour [10], while the second substage focuses on moral behaviour controlled by the adherence to the law and rules required for the maintenance of order. The post-conventional stage is the most advanced stage of moral development and one that most adults never attain. The post-conventional stage is also divided into two substages. The first substage essentially involves an altruistic outlook of care and concern for others, i.e., ‘‘an understanding of social mutuality and a genuine interest in the welfare of others’’ [11]. The second post-conventional substage of moral development in Kohlberg’s model describes individuals who have developed a belief in universal principles and personal moral control through conscientiousness derived from these principles [11].

Glenn, Iyer, Graham, Koleva and Haidt [12] point out that earlier studies of psychopath’s morality have focused on issues of justice and that Kohlbergian-oriented researchers have hypothesised that psychopaths may represent a pre-conventional stage of moral development; however, empirical results have been equivocal. Blair [13] indicates that psychopaths may have moral deficits, specifically in moral judgments involving harm, but have little difficulty in detecting conventional rule-governed transgressions (conventional level of moral development).

Blair [14] makes the important distinction between moral and conventional transgressions. A moral transgression is regarded as one that has consequences on the rights and welfare of other individuals and that while psychopaths are capable of rule-governed conventional-type behaviour, they are insensitive towards moral transgressions.

Deigh [15] suggests that the capacity for moral judgement presupposes that certain formal principles regulate practical reason. Deigh [15] maintains that psychopaths are generally incapable of:

- Universalising the intentions of moral actions to others.
- Consistently applying universalisations based on principles for all persons.

The propensity for psychopathic type behavior is also evident at the post-conventional, universalistic level of moral development, where the principles being advocated for are themselves wrong (as in the case of Nazi Germany) and inconsistently applied. This suggests that from a moral deficit point of view, psychopaths can operate at several levels of morality at the same time, with unstable mixes between higher and lower levels of moral development [9] and inconsistent integrations between idealism and relativism in their moral outlooks [1]. For this paper and the later development of a heuristic model, a moral deficit is defined as an inconsistent (i.e., idealism advocating universalistic moral principles towards some people while expressly excluding other designated people from the application of such principles) integration of idealism and relativism combined with a post-conventional level of moral development.

### 2.2. Theoretical Aspects: The Concept of Psychopathy

Psychopathy is defined by Kiehl and Hoffman [16] as:

*a constellation of psychological symptoms that typically emerges early in childhood and affects all aspects of a sufferer’s life including relationships with family, friends, work, and school. The symptoms of psychopathy include shallow affect, lack of empathy, guilt and remorse, irresponsibility, and impulsivity.*

Kiehl and Hoffman [16] also go on to point out that:

*Psychopathy is astonishingly common as mental disorders go. It is twice as common as schizophrenia, anorexia, bipolar disorder, and paranoia, and roughly as common as bulimia, panic disorder, obsessive-compulsive personality disorder, and narcissism.*

Forsyth [17] indicates that there are roughly two forms of psychopathy discernible in the literature: primary and secondary. Primary psychopathy combines emotional coldness combined with an absence of sentimentality, impulse control and altruism. Secondary psychopathy adds to these characteristics a reactive hostility and a lack of conscientiousness, which often lead to parasitic lifestyles and engaging in various forms of criminal activity to achieve their specific goals. Forsyth [17] points out that these psychopathic tendencies of lack of concern for others and compliance with social standards suggest that psychopathy negatively correlates with idealism and positively correlates with relativism. Glenn, Iyer, Graham, Koleva and Haidt [12] lend support to this contention in their comprehensive empirical study of psychopathy using several measuring instruments designed to measure moral orientations (Moral Foundations Questionnaire, Interpersonal Reactivity Index, Social Dominance Orientation, Disgust Scale, Sacredness Scale and Ethics Position Questionnaire) to investigate associations with psychopathy, as measured by Levinson’s Self-Report Psychopathy Scale (LSRP) [18].

In line with Forsyth, the study of Glenn, Iyer, Graham, Koleva and Haidt [12] indicates a negative correlation between idealism and psychopathy (r = −0.211, *p* < 0.001, n = 593) and a positive correlation between relativism and psychopathy (r = 0.214, *p* < 0.001, n = 593).

Neuroscientific brain imaging has recently been tested regarding measuring instruments of psychopathy and evidence has been found that largely supports Hare’s measurements of psychopathy with distinct neurological brain functioning in psychopaths. When summarising the evidence, Kiehl and Hoffman [16] suggest that the functioning of a psychopath’s brain is considerably different from a non-psychopath’s in neural areas of the brain that are fundamentally important in three aspects of moral judgement, namely, the ability to recognise moral issues, the ability to inhibit a response before considering its moral ramifications and the inability to reach a moral decision. References [16,19,20] have shown that each of these moral functions is associated with the paralimbic system, in which psychopaths have a marked reduction in neural activity compared to non-psychopaths.

### 2.3. Practical Aspects: Recidivism Among Psychopaths

Kiehl and Hoffman [16] indicate that psychopaths are much more likely to be recidivistic after they have been released than non-psychopaths. In 1988, Canadian researchers identified 231 incarcerated psychopaths about to be released and subjected them to assessments of psychopathy using Hare’s [21,22,23] instrument, which divided them into low, moderate and high categories of psychopathy and followed their developments for three years.

Kiehl and Hoffman’s study [16] shows that after nine months, more than half the high-score psychopaths had been rearrested and reconvicted. By the end of three years, high-psychopathy scorers had an approximately 80% recidivism rate. In comparison, only about 15% of low-psychopathy scorers had been reconvicted in the nine months since their release and only around 30% had been reconvicted at the end of the three years. 

Not all studies support the evidence that individual-level psychopathic characteristics are the only factors to consider regarding the risk of discharging purportedly rehabilitated psychopaths from psychopathic hospitals into mainstream society. Silver, Mulvey and Monahan [24] indicate that neighborhood contexts also have a predictive part to play in identifying violent recidivists. Results from their study show that neighborhood poverty has a pronounced impact in addition to the effects of individual characteristics in identifying cases with violent outcomes. Therefore, the urban locale of psychopaths who are earmarked for return to mainstream society also needs to be incorporated into the assessments and management of the risk of violence among discharged or paroled psychiatric patients.

## 3. Methodology

This study used secondary data analysis of extant literature to develop a model of a specific psychopathic “moral deficit” that appears resistant to psychiatric therapy and can lead to violent recidivist behaviour among purportedly rehabilitated psychiatric patients released into mainstream urban society. Secondary data analysis is very broadly speaking: “an analysis of data collected by someone else” [25]. Data analysis using this method involves using information from secondary data sources. The use of the method “can include any data that are examined to answer a research question other than the question(s) for which the data were initially collected” [26], as in the case of the current study. Although formal testing of hypotheses can only be carried out rigorously from primary data sources, exploratory conceptual models can be deduced from secondary data and subjected to a less rigorous form of falsification than that possible through the use of primary data. Although, as Popper [27] points out, even in the case of primary data analysis, the strength of any hypothesis depends on its degree of corroboration and this is always open and subject to falsification. Despite the explosion of secondary data and its widespread availability, it remains an underutilised scientific data resource, largely because of problems in establishing its validity and reliability. Although secondary data can be checked by the fidelity of the data’s origins (i.e., the scientific veracity its source) and the consistency with which the same data is reported by independent secondary sources, it remains more difficult to formally corroborate. The authors of the current paper did not influence the specific research designs, or the research questions asked to produce the secondary data used to build the exploratory model, which is potentially a further disadvantage of using such data for specific analysis [25].

Secondary data analysis has aspects of a historical narrative methodology in that it is qualitative and interpretative in orientation and it consists of data of past individual occurrences of connected events [28] that were collected and documented by individuals working within different knowledge paradigms (scientific, historical, journalistic) and with different objectives. The use of data in secondary data analysis is, by nature, eclectic and conjectural. As Popper [29] puts it:

*Observation is always selective. It needs a chosen object, a definite task, an interest, a point of view, a problem. And its description presupposes a descriptive language, with property words; it presupposes similarity and classification, which in their turn presuppose interests, points of view, and problems.*

Secondary data analysis is also closely related to the methodology of analytical eclecticism proposed by Sil and Katzenstein [30]. Secondary data analysis, like analytical eclecticism, generally analyses data that has already been collected and stored by someone else and uses this data to empirically verify specific hypotheses (conjectures), but not in the formal sense, which is only possible through primary data tests. In doing so, secondary data analysis is often necessarily eclectic as it focuses on specific observations from a broad and diverse range of sources that are used to explore proposed theories and models. In this form, it is qualitative and interpretative in orientation and looks for evidence that supports specific theoretical conjectures that are illustrated by specific themes that emerge from the data. Like analytical eclecticism, secondary data analysis is broadly inclusive. As Sil and Katzenstein put it:

*While research traditions generate quite varied research products—ranging from formal models and causal inferences to historical narratives and ethnographies—we follow Andrew Abbott* [31] *in viewing all of these as offering causal stories based on particular “explanatory programs”.*

A major methodological advantage, as Sil and Katzenstein [30] indicate is that with analytical eclecticism:

*the investigation of differently formulated analytic problems within contending research traditions frequently offer relevant insights for the purposes of solving substantive problems. The challenge is to compare and selectively integrate these insights so that they can be more practically useful in relation to substantive problems. Given their expanded scope, the kinds of problems addressed by eclectic scholars are more likely to have concrete implications for the messy substantive problems facing policymakers and ordinary social and political actors. (emphasis added)*

The current study uses eclectic secondary data analysis to support an exploratory theoretical model developed from interpretations of reported historical narratives of themes of recidivistic violent psychopaths showing moral deficits that combine post-conventional moral development with principles that are not considered universally applicable.

Hsieh and Shannon [32] indicate that there are three distinct ways of conducting content analysis called the conventional, directed and summative approaches. All three of the methods of content analysis interpret meaning from the content of textual data. Conventional content analysis proceeds by obtaining codes derived directly from the textual data. Directed content analysis starts with a theory or conjecture [27] and uses these to drive the search for textual data using preconceived codes in the form of keywords for guidance. The summative approach involves counting and comparisons of content and its interpretation [32]. The content approach used in the current study corresponds to the directed approach. The eclectic narrative from available secondary data sources was analysed using the directive keywords/concepts derived from the theoretical model (conjecture) [27]. These are indications in the various eclectic narratives of individual psychopathological behaviour involving psychopathy (including psychopathic tendencies), violent criminality, prior convictions of violent criminality (or individual psychopathology indicative of this possibility), recidivism (after purportedly successful psychiatric therapy), and most importantly, “moral deficit” (involving indications of idealist principled levels of moral development with relativistic exclusions of specific groups. The strategy for the directed content analysis used in the current study essentially conforms to that suggested by Hsieh and Shannon [32], which is to begin coding and interpreting the data with the above predetermined codes. However, as Hsieh and Shannon [32] point out:

*The directed approach does present challenges to the naturalistic paradigm. Using theory has some inherent limitations in that researchers approach the data with an informed but, nonetheless, strong bias. Hence, researchers might be more likely to find evidence that is supportive rather than non- supportive of a theory.*

Furthermore, overemphasis on theory can make the researcher blind to differing contextual aspects [32]. Despite these shortcomings, directed content analysis can provide a useful preliminary step in building exploratory theoretical models that can be, if tentative support is found for them, subsequently exposed to empirical tests.

## 4. Results: Eclectic Psychiatric Reports, Historical Narrative and Rehabilitation Research

This section discusses two specific aspects. The first subsection describes eclectic secondary data narratives obtained from available sources that illustrate the behaviour of violent terrorists with psychopathic tendencies and suggests specific moral deficits that are used to build the exploratory model. The second subsection briefly describes recent extant research findings describing the practical problem of psychopathic therapy and rehabilitation. This subsection indicates how high measured levels of psychopathy with specific moral deficits resist attempts at rehabilitation because they are unable to target specific deficits. The exploratory model discussed in Section 4 of the paper aims to provide a theoretical heuristic that targets a psychologically measurable specific moral deficit associated with violent psychopathy (as indicated from the eclectic secondary data narratives) to reduce the occurrence of violent psychopathic recidivistic behaviour in the urban environment in particular.

### 4.1. Eclectic Secondary Data: Violent Terrorists with Psychopathic Tendencies

There is a paucity of detailed secondary data available on individual historical narratives of violent recidivistic psychopathic behaviour for three main reasons. First, it is only reported in the media when it occurs, which is relatively rare. Second, detailed material is often withheld from the general public for socio-political reasons. Third, and perhaps most importantly, detailed psychiatric material is not generally available as it is confidential patient material. Although the secondary data discussed in the following material is eclectic (i.e., obtained from a broad and diverse range of potentially available narratives), much potential material remains unavailable for research purposes. For this reason, secondary data from two major case narratives with substantial psychiatric evidence available in published material and a third example (with little psychiatric evidence available) of the case of a recidivist Islamic terrorist with psychopathic tendencies who had successfully conned therapists that he had been successfully rehabilitated and was “safe” to return to the London urban environment, were used to build the exploratory model. These cases aim to illustrate the problem of post-conventional-type moral belief systems combined with a dysfunctional idealist and relativistic outlook, which are postulated in this paper as being a source of “moral deficit” found in violent recidivistic terrorists with psychopathic tendencies and in violent psychopaths. Schuurman [33] points out that the view that terrorists are psychopathic has been disputed in the literature and clearly not all terrorists are psychopathic. However, it is also probably true to say that given the difficulties in defining and measuring psychopathy, there are many terrorists with psychopathic tendencies, or in other words, who display some of its core characteristics [32]. The main issue with this argument is the heterogeneity of the terrorist. It is important to distinguish mental illness from a personality disorder. Mental illnesses, such as depression, post-traumatic stress disorder (PTSD) and schizophrenia, affect a person’s ability to function, whereas a personality disorder is not necessarily “dysfunctional” as an individual with a personality disorder can be highly functioning and their volatility in pursuing self-interest makes them difficult to control and a likely risk to terrorist groups [33]. Furthermore, it is difficult to complete a clinical diagnosis of terrorists. If the literature cannot determine a terrorist profile or personality, it might be argued that “some” terrorists have psychopathic traits and others suffer a mental illness that may contribute to their argued moral incapacity. However, to argue this from the perspective of individuals with a diagnosed mental illness like schizophrenia is complex. Symptom presentation for schizophrenia is primarily psychosis, a loss of touch with reality. This manifests in hallucinations (auditory, visual, taste or smell) and delusions, such as delusions of grandeur (feeling as though you are godlike or have a supreme talent) or persecutory delusions (feeling as though you are being watched/stalked) [33].

Although not all terrorists are regarded as psychopathic, there are several cases of recent violent terrorism perpetrated by persons with psychopathic tendencies [33] who appear to present a “moral deficit” through a specific moral problem that combines high levels of moral development with an unstable idealist and relativist integration [9,12,17]. The terrorist case of Anders Breivik is a recent example of terrorist behaviour that bears aspects of this specific “moral deficit” that requires further detailed discussion and analysis. Wollenberg [34] describes the horrific case of Breivik as follows:

*On 22 July 2011, at 3:35 p.m., Anders Behring Breivik set off a car bomb in front of the office of Prime Minister Jens Stoltenberg and other government buildings in Oslo. The explosion killed eight and wounded over 200 people. Less than two hours later, at a summer camp on the island of Utøya run by the Workers’ Youth League, the youth division of the ruling Norwegian Labor Party, Breivik, wearing a homemade police uniform, opened fire, killing 69 and wounding 110, mostly teenagers.*

Breivik was born in Oslo in 1979. His father worked for the Norwegian foreign services. His parents divorced in 1980 and he grew up with his mother and limited contact with his father. His mother requested help from the Child Welfare Services twice because he was a difficult child. Breivik was examined and assessed by the Child Psychiatric Services in 1983. The Child Psychiatric Service found that the way he was being brought up by his mother was problematic and that he was in danger of developing severe psychopathology. The Child Psychiatric Service recommended that he should be committed to foster care. However, the Child Welfare Services decided against this action, and after supervising Breivik at home for a short period, his case was closed in 1984 [35]. Thus, although the possibility of Breivik developing severe psychopathy was identified early on by the Child Psychiatric Service, he was returned to his mother and a problematic upbringing that resulted in a level of urban violence and the loss of innocent lives never seen before in the Norwegian urban environment.

Breivik wrote a 1500-page manifesto outlining his extreme negative views on multicultural societies, Islam and Marxism, and posted it online on 22 July 2011 [35]. The manifesto, although extremist in tenor and indicated his inconsistent idealist and relativist values [1,2,17], suggested a post-conventional level of moral development in the moral principles being advocated [9].

After the terrorist acts and his arrest and detainment by Norwegian police, two psychiatrists conducted 36 h of investigation with Breivik and his interrogation by police, along with interviewing his mother. After conducting a series of unstructured and diagnostic structured interviews using accepted psychiatric procedures, the psychiatrists reported that Breivik was psychotic when planning and implementing his terrorism. Psychiatrists regarded him as being affectively deficient but with no indications of depression, mania or auditory hallucinations [34].

In 2013, two more psychiatrists were consulted and they diagnosed Breivik as not being psychotic but instead had a severe narcissistic personality disorder and a pathological lying tendency. The psychiatrist maintained that Breivik was therefore legally responsible for his terrorist acts of murder. These diametrically different diagnostic outcomes by professional psychiatrists indicate the difficulties in reaching psychiatric conclusions regarding violent acts of terrorism [33,34]. However, it is clear from Breivik’s case history that he was not psychotic but rather possessed psychopathic traits (e.g., pathological lying and stunted affectivity) [21,22] with a post-conventional level of moral development [9] since he believed strongly in specific, albeit extremist, principles regarding Islam, Marxism and in the preservation of Western culture. He also displayed an idealism–relativism mix that advocated an inconsistent integration of universal principles, such as Western cultural preservation with a relativist intolerance of Islam [1,2,17].

A further case study of violent behaviour in an individual who appeared to have a high level of moral development combined with an unstable and inconsistent integration of idealism and relativism is that of Mohammed Bouyeri, a second-generation Moroccan-Dutchman [36]. He studied for a degree, changing his major several times, but was unable to complete it. He was known to Dutch police as a “problem youth” [36]. In 2003, after his mother died and father remarried, he started living in accordance with Sunni Islamic Sharia principles. Bouyeri’s murder victim was Theo Van Gogh, a filmmaker critic of religion and Islam in particular. He directed a short film, “Submission”, about Islamic violence towards women. Bouyeri killed van Gogh on 2 November 2004 while he was bicycling to work. He shot van Gogh eight times and wounded two bystanders. After being shot, van Gogh ran to the other side of the road and fell to the ground. van Gogh’s last words were reputed to have been asking Bouyeri for mercy. Bouyeri subsequently cut van Gogh’s throat, attempted to decapitate him and then drove a large knife deep into his chest. Bouyeri was arrested close to the scene of the crime after an exchange of gunfire with police that including Bouyeri being shot in the leg [36].

In the interrogations following his arrest, he exercised his legal right to remain silent. When Bouyeri was arrested he was found to have a poem that suggested that he wanted to die a martyr. During his trial for terrorism and murder, Bouyeri carried a copy of the Quran. He showed no remorse for the murder he admitted to committing, saying to van Gogh’s mother: “I do not feel your pain. I do not have any sympathy for you. I cannot feel for you because I think you are a non-believer.” [36] Bouyeri also maintained that “in the fight of the believers against the infidels, violence is approved by the prophet Muhammad” [36]. On 26 July 2005, Bouyeri was sentenced to life in prison, which is the most severe punishment under Dutch law and presents no chance of parole [36].

Although psychiatric material has not been published on the case, Bouyeri showed clear psychopathic tendencies of affective deficiency and absence of remorse [21,22]. His belief in Islam and Islamic principles suggests he had a post-conventional level of moral development [9] but mixed the universalism of his idealism with relativism [1,2] in displaying no mercy while killing a non-believer of Islam.

After Bouyeri’s terrorism, Islamic terrorist activity has become more pronounced. During the period 2014–16, there were more people killed by Islamic terrorist attacks in Europe than all previous years combined, where most of this more recent terrorist activity was inspired by militants of the Islamic State (ISIL) [37,38]. Bartholomew [39] notes that:

*The year 2015 saw an upsurge in terrorist attacks on Western countries that were reportedly inspired by Islamic State in Iraq and Syria (ISIS). A number of less serious incidents have also occurred including the stabbing of three passengers on the London Underground rail network on 5 December.*

A very recent example of Islam-inspired terrorism in the urban environment by a convicted terrorist with psychopathic tendencies who had been prematurely released back into urban society is presented in the case of Kahn in London [40,41]. No detailed psychiatric evidence is available on the case of Khan, but it shows how a terrorist with clear psychopathic traits of deception and manipulation could evade detection by pretending to have been effectively rehabilitated by extant therapy [40,41]. Here, again, there is circumstantial evidence that Kahn may have had a post-conventional belief [9] in the moral principles of radical Islam while also displaying an inconsistent integration between idealist universal beliefs with the relativism of the universal principles [1,2], advocating that they were only relevant and applicable to believers of Islam and claiming they actively justified the killing of non-believers.

The London Bridge attack occurred on 29 November 2019, but Khan’s path to radical Islamic belief started when he was 14 years old when he became active in the British AI Muhajiiroun Islam extremist network. At 16, he became involved with the radical extremist preacher, Anjem Choudary, who was released from prison to home arrest in 2019 [41]. Khan came to the attention of British security after joining a group of eight Al Qaeda extremists who were engaged in designing bomb attacks throughout the UK and building terrorist training facilities. Khan was convicted of terrorism in 2012 and was imprisoned for eight years. He was released in 2019 and lived in a probation hostel in Staffordshire. He was required to wear an electronic ankle bracelet that allowed police to track his whereabouts at any time. He had restrictions on his phone and was required to meet with a probation officer twice a week [41]. Kahn had been exposed to the government’s Desistance and Disengagement, which was a holistic rehabilitation program that included mentoring, psychological counselling and theological and ideological advice. Khan was also enrolled in the government’s counterterrorism Healthy Identity Program, and during the program, had written extensive reports on his progress maintaining that he was no longer a threat to society [41]. In doing so, Khan demonstrated typical psychopathic traits of pathological lying and manipulation to con the authorities [21,22]. Khan had been under MI5 surveillance since 2018 and was considered a “low-to-medium risk”. On the day of the killings, he had attended a conference where he spoke of his rehabilitation, which another conference participant described as a “compelling success story”. During a break in the conference programme, Khan went to the toilet and came out wearing a fake suicide vest and had two knives taped to his wrists. He then set out on his killing spree and killed two Cambridge University graduates who were attendees at the conference and injured three others. He was shortly thereafter apprehended by police and shot dead [41].

The Khan case clearly indicates the problem of allowing inmates with psychopathic tendencies and specific moral deficits, and who had given every indication of successful rehabilitation, back into society.

In general, the results of all three secondary data narratives clearly indicate aspects of the concepts used to perform the directed content analysis. The Breivik narrative shows the case narrative of a man who had an early psychopathological history (although he had not been incarcerated and had not received specific remedial therapy). His psychiatric reports suggest that he had multiple psychopathic characteristics and the data in the narrative suggests he may have combined a highly developed post-conventional moral outlook [9] with idealistic principles and a relativistic [1,2,4] approach that excluded Marxists and leftists who advocated multiculturalism and Muslims from these universalistic principles. He committed devastatingly violent criminal acts before being apprehended on those he perceived as representing a section of society to be expunged partly (as argued in the current paper) based on his morally defective mix of idealism and relativism.

The case of Bouyeri presents a similar narrative of a man who had been regarded as a “problem youth” by Dutch police [36] but had not been imprisoned or received specific remedial therapy. Although specific psychiatric reports are unavailable, statements Bouyeri made during his trial clearly suggest psychopathic characteristics of lack of remorse and affective deficiency [21,22]. He also seemed to combine a post-conventional, principled level of moral development [9] with idealistic, albeit extremist, views of Islam, which advocated the relativistic [1,2,4] belief of the necessity of the violent removal of infidels from society, such as van Gogh, whom he murdered.

Finally, the recent case of the London Bridge attack offers a narrative of violent psychopathic behaviour of a man who had been imprisoned as a terrorist for eight years and had received specific remedial therapy [40,41]. The case of Khan is of special interest because he had received specific therapy to rid him of his extremist Islamic beliefs and had been released on probation into urban society on the supposition that he had been successfully treated and was no longer a threat [41]. Khan had also shown characteristic psychopathic traits of pathological lying and manipulation, and after his release on probation, he had gone on a knife-wielding killing spree that cost the lives of two graduates and injured three others. Here, as in the other cases, a seemingly post-conventional level of moral development [9] in the principles of Islam is combined with a relativistic [1,2,4] exclusion of these idealistic principles regarding infidels and the justification of killing such unbelievers.

### 4.2. Psychopathic Therapy and Rehabilitation

Kiehl and Hoffman [16] indicate that:

*Law and psychiatry, even at the zenith of their rehabilitative optimism, both viewed psychopaths as a kind of exception that proved the rehabilitative rule. Psychopaths composed that small but embarrassing cohort whose very resistance to all manner of treatment seemed to be its defining characteristic.*

One reason for this resistance may be that existing therapy does not focus on dysfunctional aspects in psychopathic moral processing. For example, Kuzhoharova et al.’s [42] research concerning impairments of the threat process in psychopaths focus on two factors in psychopathy: factor 1, “affective and interpersonal deficits”, capture affective deficits, such as a lack of empathy that characterises psychopathic cognition and is a core aspect of their “moral deficit”; and factor 2, interpreted as “antisocial and impulsive/disorganised behaviour” and an embraced life-course of persistent antisocial behaviours. Kuzhoharova et al.’s [42] research found a higher score of impaired processing of threat for factor 1. This idea of impaired threat processing can be regarded as a consequence and/or ramification of the psychopath’s moral deficit in factor 1. Kuzhoharova et al. [42] maintain that:

*Traditional treatments within the criminal justice system are relatively ineffective for psychopathic offenders. One possible explanation is that these treatments do not address the unique patterns of dysfunctions present in psychopathic individuals. Findings that the two factors are associated with distinctive cognitive-affective functions, from our studies and others, strongly suggest that developing evidence-based treatments depends upon targeting the unique factor-specific deficits. Directly translating the current results into clinical practice would suggest that individuals with higher scores on Factor 1 will not be able to utilize aversive learning to shape behaviour, and so alternative strategies are required.*

However, Kuzhoharova goes on to state: “Cognitive remediation training targeting the dysfunctions associated with the two factors have shown promising preliminary results” [42,43].

Caldwell [44] and colleagues, based at the University of Wisconsin, reviewed available treatment literature and decided to design a program for the treatment of juvenile psychopathic offenders. Caldwell borrowed from a large array of treatment theories and methods to build a new “decompression” treatment. The treatment was intense, lasting several hours daily and had a long duration lasting six months to a year of one-on-one rebuilding of the social networks and affectivity missing in psychopaths.

In a pilot study of 30 violent juvenile offenders, Caldwell [44] divided them into three groups of 10 people. In the two control groups used, one group received no therapy, the second group received traditional group therapy, while the treatment group received Caldwell’s decompression therapy. The study had a follow-up period of two years and the recidivism findings were encouraging: 70% of the control group who did not receive treatment were recidivistic at least once during the two years compared to 20% of the group that received traditional therapy and 10% of the group subjected to decompression treatment.

In a later study, Caldwell and his co-researchers [45] followed the behaviour of 86 maximum-security juvenile defenders who had been assessed for psychopathy using Hare’s measuring instrument for juvenile offenders, namely, the Psychopathy Check List: Youth Version (PCL-YV), which is a quantitative measure of institutional misconduct, security days (SD), and re-arrest data.

The findings of the study indicated that the PCL-YV scores were high (mean = 30.2) and were strongly correlated to recidivism and SD. Second, the decompression treatment was found to be highly effective in reducing SD and recidivism if it was applied over a long period and for psychopaths scoring in the low to moderate range of the PCL-YV (<31). However, shorter-term decompression was found to have little effect on SD and recidivism among juvenile psychopaths.

## 5. The Development of an Exploratory Model of Specific Violent Psychopathic-Type Individuals with Low Effective Therapy and High Recidivism Propensities

**Figure 1:**
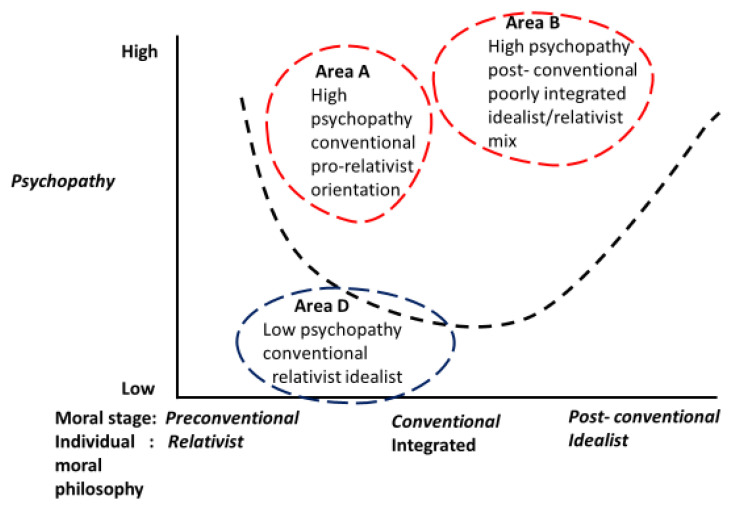
An exploratory model of specific violent psychopathic-type individuals with low effective therapy and high recidivism propensities.

Figure 1 presents a diagrammatic representation of the exploratory model of a specific form of moral deficit that we maintain may act as an additional predictor of violent recidivistic behaviour when found in individuals with high psychopathic tendencies [21,22], which as we have seen, are also associated with high levels of violent recidivism and low levels of effective rehabilitation using various forms of therapeutic intervention. The exploratory model aims to provide a further therapeutic possibility from the measurement of a specifically targeted psychopathic moral deficit [42,43] consisting of post-conventional levels of moral development [9] with an incapacity to consistently apply universalised idealistic [1,2,3,4] moral principles to all persons [15].

In Figure 1, the vertical axis indicates the extent of psychopathy (low to high) as measured by conventional extant measuring instruments [18]. The horizontal axis measures the level of moral development, as defined by Kohlberg [9], and levels of relativism, idealism and relativism/idealism [1,2,4] integration in an individual’s moral philosophical outlook [17]. The exploratory model postulates that the sketched areas A and B are areas of high measured psychopathy [18], which recent research [16] has shown to be associated with violent criminal behaviour, low levels of effective rehabilitation and high levels of recidivism [16,44]. Area A, in line with Caldwell’s [44] study, indicates that individuals with high psychopathy scores are less amenable to decompression therapy. Such difficult-to-rehabilitate, high psychopathy scorers are indicated in Figure 1 as having a conventional level of moral development [9]. This seems reasonable since it has been found that most psychopaths have been shown to have little difficulty with rule-governed activity [46], which is characteristic of the conventional level of moral development [9], and have pro-relativist [1,2,17] moral profiles. In other words, as indicated in Figure 1, this suggests that persons who score high on measured psychopathy [18] and who have a moral profile consisting of a conventional level of moral development and a relativistic moral profile [1,2,17] will be more difficult to rehabilitate [44]. This aspect has both empirical support and is consistent with the exploratory model displayed in Figure 1, as well as Caldwell’s [44] separate study that found high psychopathy scorers (as measured by various psychological and psychiatric instruments [18]) were associated with problems of rehabilitation and recidivism.

Area B corresponds to the postulated relationship built from the eclectic secondary data, which we are proposing as a novel theoretical contribution in the current study that suggests a further aspect of psychopathic moral deficit that consists of:

- Post-conventional levels of moral development [9].
- Inconsistent integrations of idealism and relativism [1,2].
- High levels of measured psychopathy and violent criminal behaviour.
- Poor prospects of rehabilitation through extant therapeutic practices.

The latter two points are made clear in Caldwell and van Rybroek’s study [44], which indicates that juvenile inmates with high psychopathy scores and recidivistic tendencies are less amenable to therapeutic interventions, including decompression therapy [43]. Juvenile inmates with low psychopathy scores (area D in Figure 1) were found by Caldwell and van Rybroek [44] to be associated with lower recidivism and more effective rehabilitation after receiving compression therapy. In line with Caldwell and van Rybroek’s findings, it follows that individuals with high psychopathy and a moral deficit (as defined in the current study) are likely to be prone to violent recidivistic behaviour, and by implication, also less amenable to decompression therapy.

## 6. Discussion

Recent terrorist violent crime by persons with psychopathic characteristics who appear to have the unusual admixture of integrated idealism and relativism, alongside post-conventional levels of moral development, who have not been appropriately treated by extant psychiatric therapy or who have escaped identification entirely are illustrated in by the cases of Bouyeri, van Gogh’s assassin; Kahn, the Muslim extremist, who when released on parole, subsequently embarked on a killing spree in London; and Breivik, who was responsible for the deaths of 77 innocent people. All these cases described in the paper exemplify the same admixture of high moral development and an inconsistent integration of idealism with relativism, which also appeared to reflect the underlying individual moral outlook of Nazi leaders and their followers that motivated the Nazi holocaust discussed earlier.

The recent cases of recidivist psychopaths, or those who are clearly mentally ill but may not have been classified formally as psychopaths, suggests that despite the sophistication of extant psychiatric therapy and its success in some instances, aspects of psychopathy diagnosis and identification are clearly failing and are making the urban environment unsafe. The paper suggests that a common denominator in specific violent psychopaths released after rehabilitation is a moral deficit of the form described in the paper. In fact, the outlined extant data suggests, in line with the exploratory model and thus adding to its plausibility, that high measured psychopathy, a conventional level of moral development and a pro-relativistic moral outlook can be found in psychopaths who are resistant to conventional therapy.

Limitations of the study include the fact that the secondary data and narrative material and reports used to build the model are eclectically drawn from a broad base of diverse sources and may thus postulate an association between a specific type of moral deficit and violent psychopathy that may turn out to be false (a type 1 error). In this regard, Berg and Lane [47] indicate that a major limitation of historical narrative research is its internal validity, in other words, there are concerns regarding whether a specific historical secondary source shows what it purports to show. This problem can often be counteracted by the consistency of the data reports documented [48,49]. However, in the case of the current study, the paucity of the availability of extant secondary data arose at least partly from the fact that much of the data about violent psychopathic behaviour (especially individual psychiatric and psychological cases) are not widely available. Furthermore, the study is theoretically oriented and exploratory, and the model is tentative and is open to falsification through primary data analysis. Moreover, in the identification and diagnosis of psychopathic behaviour and its multifaceted nature, particularly of violent recidivistic psychopathy, there are clearly many causal factors involved and other psychological, psychiatric and neurological instruments that can be used to measure its incidence and occurrence. Whether the conjectures made in the description of the exploratory model are found to be a useful additional tool in the already available formidable arsenal for the identification of high risk recidivistic violent psychopathy remains an open question. However, it should be emphasised that the paper is not aimed at providing a mono-causal theory of violent psychopathy and recognises the tentative nature of the model presented and the fact that it can at best provide only a small contribution to the sophisticated armory of knowledge already available on the topic.

## 7. Conclusions

There is evidence that the problem of moral deficit in psychopathic behaviour has not received the attention it deserves in the extant literature. The paper has tried to show, through an exploratory model built from eclectically sourced secondary data and the analysis of selected psychiatric, psychological and media historical narratives of recent cases of violent criminality in our cities, that a specific kind of moral deficit, which has not been fully identified in psychopaths and which may be resistant to psychiatric therapy, could result in morally unbalanced individuals being prematurely released into our cities with devastating consequences. The practical implications of the tentative model, if corroborated through primary research data, are that psychopaths who have the specific moral deficit proposed in the model should be monitored closely and possibly offered extended specialised therapy of the kind proposed by Caldwell [43] before being allowed back into mainstream society.

To conclude, the exploratory model has aimed to provide a coherent conceptual argument for the development of empirical propositions for testing and analysis. It is recommended that future research is done using the exploratory model to undertake empirical tests using primary data to investigate its reliability and validity further.

## References

[1] Forsyth D.R. (1980). A taxonomy of ethical ideologies. J. Pers. Soc. Psychol..

[2] Forsyth D.R. (1985). Individual differences in information integration during moral judgment. J. Pers. Soc. Psychol..

[3] Forsyth D.R. (1992). Judging the morality of business practices: The influence of personal moral philosophies. J. Bus. Ethic..

[4] Forsyth D.R., Kurtines W.M., Azmitia M., Gewirtz J.L. (1992). Values, conceptions of science, and the social psychological study of morality. The Role of. Values in Psychology and Human Development.

[5] Schlenker B.R., Forsyth D.R. (1977). On the ethics of psychological research. J. Exp. Soc. Psychol..

[6] Bialas W., Fritze L. (2014). Nazi Ideology and Ethics.

[7] Wachsmann N. (2015). KL: A History of the Nazi Concentration Camps.

[8] Mineau A. (2007). Himmler’s ethics of duty: A moral approach to the Holocaust and to Germany’s impending defeat. Euro. Leg..

[9] Hanford J.T., Kohlberg L. (1982). The Philosophy of Moral Development. J. Sci. Study Relig..

[10] Carroll A.B., Brown J.A., Buchholtz A.K. (2018). Business & Society: Ethics, Sustainability, and Stakeholder Management.

[11] Barger R.N. Philosophical Belief Systems. Retrieved 9 September 2009. http://www.nd.edu/~rbarger/philblfs.html.

[12] Glenn A.L., Iyer R., Graham J., Koleva S., Haidt J. (2009). Are all types of morality compromised in psychopathy?. J. Pers. Disord..

[13] Blair J. (2007). The amygdala and ventromedial prefrontal cortex in morality and psychopathy. Trends Cogn. Sci..

[14] Blair R. (1995). A cognitive developmental approach to morality: Investigating the psychopath. Cognition.

[15] Deigh J. (1995). Empathy and universability. Ethics.

[16] Kiehl K.A., Hoffman M.B. (2011). The criminal psychopath: History, neuroscience, treatment, and economics. Jurimetrics.

[17] Forsyth D.R. (2020). Making Moral Judgements: Psychological Perspectives on Morality, Ethics and Decision-Making.

[18] Levenson M.R., Kiehl K.A., Fitzpatrick C.M. (1995). Assessing psychopathic attributes in a noninstitutionalized population. J. Pers. Soc. Psychol..

[19] Blair R.J.R. (2003). Neurobiological basis of psychopathy. Br. J. Psychiatry.

[20] Raine A., Buchsbaum M., Lacasse L. (1997). Brain abnormalities in murderers indicated by positron emission tomography. Boil. Psychiatry.

[21] Hare R.D., Clark D., Grann M., Thornton D. (2000). Psychopathy and the predictive validity of the PCL-R: An international perspective. Behav. Sci. Law.

[22] Hare R.D., Neumann C.S., Patrick C. (2006). The PCL-R assessment of psychopathy: Development, structural properties, and new directions. Handbook of Psychopathy.

[23] Hare R.D. (1991). The Psychopathy Checklist-Revised.

[24] Silver E., Mulvey E.P., Monahan J. (1999). Assessing violence risk among discharged psychiatric patients: Toward an ecological approach. Law Hum. Behav..

[25] Boslaugh S. (2007). Secondary Data Sources for Public Health.

[26] Vartanian Y.P. (2011). Secondary Data Analysis.

[27] Popper K.R. (1972). Objective Knowledge: An. Evolutionary Approach.

[28] Payne M., Barbera J.R. (2013). Dictionary of Cultural and Critical Theory.

[29] Popper K.R. (2002). Conjectures and Refutations: The Growth of Scientific Knowledge.

[30] Sil R., Katzenstein P.J. (2010). Analytic eclecticism in the study of world politics: Reconfiguring problems and mechanisms across research traditions. Perspect. Politi..

[31] Abbot A. (2004). Methods of Discovery: Heuristics for the Social Sciences.

[32] Hsieh H.F., Shannon S.E. (2005). Three approaches to content analysis. Qual. Health. Res..

[33] Schuurman B. (2018). Research on Terrorism, 2007–2016: A Review of Data, Methods, and Authorship. Terror. Political Violence.

[34] Wollenberg D. (2014). The new knighthood: Terrorism and the medieval. Postmedieval.

[35] Melle I. (2013). The Breivik case and what psychiatrists can learn from it. World Psychiatry.

[36] Browne A. (2005). I Feel no Sympathy for You: Killer Tells van Gogh Family. The Times.

[37] Nesser P., Stenersen A., Oftedal E. (2016). Jihadi terrorism in Europe: The IS-effect. Perspect. Terror..

[38] Hughes S. (2017). Allies under attack: The terrorist threat to Europe. Program. on Extremism.

[39] Bartholomew R.E. (2016). The Paris terror attacks, mental health and the spectre of fear. J. R. Soc. Med..

[40] Grierson J. (2019). Islamic Extremists Remains Dominant UK Terror Threat, Say Experts. The Guardian.

[41] Duncan P., Stubley P. (2019). London Bridge Attack: First Victim Named as Pressure Mounts on Johnson for Investigation into Release of Convict Taught by Anjem Choudary. The Guardian.

[42] Kozhuharova P., Dickson H., Tully J., Blackwood N. (2019). Impaired processing of threat in psychopathy: A systematic review and meta-analysis of factorial data in male offender populations. PLoS ONE.

[43] Baskin-Sommers A.R., Curtin J.J., Newman J.P. (2015). Altering the cognitive-affective dysfunctions of psychopathic and externalizing offender subtypes with cognitive remediation. Clin. Psychol. Sci..

[44] Caldwell M.F., Van Rybroek G.J. (2001). Efficacy of a decompression treatment model in the clinical management of violent juvenile offenders. Int. J. Offender Ther. Comp. Criminol..

[45] Caldwell M., McCormick D.J., Umstead D., Van Rybroek G.J. (2007). Evidence of treatment progress and therapeutic outcomes among adolescents with psychopathic features. Crim. Justice Behav..

[46] Cima M., Tonnaer F., Hauser M.D. (2010). Psychopaths know right from wrong but don’t care. Soc. Cogn. Affect. Neurosci..

[47] Berg B.L., Lune H. (2012). Qualitative Research Methods for the Social Sciences.

[48] Kipping M., Wadhwani R.D., Bucheli M. Analysing and Interpreting Historical Sources: A Basic Methodology. https://www.researchgate.net/publication/301093205_Analyzing_and_Interpreting_Historical_Sources_A_Basic_Methodology.

[49] Stinchcombe A.L. (2005). The Logic of Social Research.

